# Supplementary material for: Genome-Wide Analysis of the Temporal Genetic Changes in Streptococcus pneumoniae Isolates of Genotype ST320 and Serotype 19A from South Korea
Source: Microorganisms. 2021 Apr 10;9(4):795. doi: 10.3390/microorganisms9040795 (PMC8069037; doi:10.3390/microorganisms9040795)
Supplement: Supplementary file 1 [file microorganisms-09-00795-s001.pdf]

Table S1. The whole-genome sequencing statistics of 26 *S. pneumoniae* strains in this study.

Strain	No. of contigs	Total length (bp)	GC content (%)
99-176	1	2,089,994	39.84
99-192	23	2,045,219	39.75
04-041	24	2,026,658	39.76
04-177	29	2,043,139	39.75
05-384	28	2,045,894	39.75
05-404	27	2,044,745	39.74
06-101	24	2,042,266	39.75
06-300	28	2,078,367	39.76
07-028	27	2,043,298	39.75
07-093	23	2,024,358	39.83
08-087	25	2,045,883	39.75
08-114	22	2,028,603	39.76
09-125	28	2,039,966	39.76
09-145	25	2,043,088	39.75
10-058	28	2,046,303	39.75
10-287	24	2,043,734	39.75
11-138	30	2,045,375	39.75
11-194	24	2,040,602	39.77
12-039	29	2,045,809	39.74
12-102	25	2,042,173	39.75
13-075	27	2,040,359	39.76
13-103	27	2,033,125	39.75
14-109	28	2,083,098	39.78
14-212	29	2,043,076	39.75
15-123	23	2,040,596	39.76
15-019	237	2,329,094	39.52
